# The impact of mode of subsequent birth after obstetric anal sphincter injury on bowel function and related quality of life: a cohort study

**DOI:** 10.1007/s00192-020-04234-3

**Published:** 2020-02-24

**Authors:** Sara S. Webb, Alice Sitch, Christine MacArthur

**Affiliations:** 1 Birmingham Women’s & Children’s NHS Foundation Trust, Edgbaston, Birmingham UK; 2 Institute of Applied Health Research, College of Medical and Dental Sciences, University of Birmingham, Edgbaston, B15 2TT UK; 3 NIHR Birmingham Biomedical Research Centre, University Hospitals Birmingham NHS Foundation Trust and University of Birmingham, Birmingham, UK

**Keywords:** Quality of life, Bowel function, Obstetric sphincter injury, Subsequent birth

## Abstract

**Introduction and hypothesis:**

The objective was to assess the impact of mode of subsequent birth on bowel function and related quality of life (QoL) in pregnant women with previous obstetric anal sphincter injury (OASI).

**Methods:**

A prospective cohort study, designed, undertaken and reported using the Strengthening the Reporting of Observational Studies in Epidemiology statement and checklist. All pregnant women with previous OASI recruited at a specialist antenatal OASI clinic in a tertiary hospital to discuss mode of subsequent birth, between 1 January 2014 and 31 October 2015. Women are counselled in line with local guidelines based on Royal College of Obstetricians and Gynaecologists Green-top recommendations. In addition to routine endoanal ultrasound scan (EAUS), women recruited to the study were asked to complete the validated Manchester Health Questionnaire (MHQ) at both 34 weeks’ gestation and 6 months postnatally.

**Results:**

Of the 175 study participants, 125 (71.4%) completed follow-up at 6 months. There was no significant change in frequency of bowel symptoms or QoL domain scores in women who had a subsequent vaginal birth compared with caesarean section. Multivariate analysis showed the odds of having poor “incontinence impact” (OR 2.91, 95% CI 1.03–8.21) and “physical limitations” (OR 4.56, 95% CI 1.02–20.45) were significantly higher for women who had a subsequent caesarean section.

**Conclusions:**

For women with previous OASI, a subsequent vaginal birth is suitable for those with no bowel symptoms and normal EAUS and caesarean section is reasonable for women who do not have normal bowel function *and/or* normal EAUS findings; however, for some of these women bowel symptoms and QoL may be worsened.

**Electronic supplementary material:**

The online version of this article (10.1007/s00192-020-04234-3) contains supplementary material, which is available to authorized users

## Introduction

Obstetric anal sphincter injuries (OASIS) are serious complications of vaginal birth with a reported UK incidence of 5.9%, with UK data demonstrating a tripling of incidence over the past decade, possibly because of increased awareness and improved methods of detection [1]. OASIS are recognised to be a major risk factor for anal incontinence (AI), resulting in concern among many of the women who have previously sustained an OASI when considering the mode of birth for a subsequent pregnancy.

Since the introduction of the Royal College of Obstetricians and Gynaecologists (RCOG) Green-top Guideline “The Management of Third- and Fourth-Degree Perineal Tears in 2001” [2], the repair and immediate management of women who sustain OASIS has been standardised and improved. However, the recommendations on mode of birth for women who have a subsequent pregnancy are not based on good evidence from research specifically designed to investigate the effect of mode of subsequent birth on bowel function and quality of life in women with a previous OASI [3].

The few studies that have considered bowel function following a subsequent birth have been service evaluations of local policy guidelines [4–9]; some also restricting inclusion criteria to women who had subsequent vaginal birth only [5] or excluding women who experienced a subsequent birth mode that was “against” recommendation, whether through maternal choice or natural events [4, 7]. Service audits necessarily exclude women who do not return to the specialist clinic in the postnatal period and such women are likely to be systematically different, thus introducing bias. Moreover, given that actual mode of birth sometimes differs from planned mode, it is important that such women are not excluded. There have also been few studies looking at QoL, despite evidence that for women with a previous OASI even mild bowel symptoms can affect the women’s quality of life in the longer term [10].

The aim of this research was to assess the impact of the mode of subsequent birth on bowel function and related QoL in pregnant women who had previously sustained an OASI, counselled as per current RCOG Green-top Guideline recommendations, in a high-quality cohort study [11].

## Materials and methods

A prospective cohort study of all eligible pregnant women with a previous OASI recruited at the specialist antenatal OASI clinic at Birmingham Women’s and Children’s NHS Foundation Trust, Birmingham, UK, to discuss the mode of the subsequent birth, between 1 January 2014 and 31 October 2015. This is a tertiary teaching hospital with approximately 9,000 births per annum. Women were eligible unless they were under 16 years of age or could not read or write in English. This was necessary because the bowel function and QoL outcome measures were collected using self-completed questionnaires that were validated for use in English only.

In line with routine hospital protocol based on the RCOG Green-top Guideline [11], all pregnant women with a previous OASI were reviewed at 30–34 gestational weeks by the specialist perineal midwife so that endoanal ultrasound (EAUS) could be undertaken, if not previously done following the OASI, and to discuss and plan the mode of birth for the current pregnancy. Anorectal manometry is not undertaken for women with a previous OASI as the published “normal” range is derived from studies using a heterogeneous population of a wide age range, including men, women and incontinent subjects. There is currently very little published research on squeeze pressures for pregnant women [12]; therefore, the use of anal manometry as a diagnostic tool to identify abnormal bowel function in women during the antenatal and postnatal period remains subjective.

Anal sphincter integrity was determined by endoanal scan images undertaken on a BK 5052 machine using an oil-filled rotating endoprobe. A defect in the external anal sphincter was diagnosed by a hypoechoic area where the muscle is disrupted, either full or partial thickness and/or an area of excessive scarring greater than 30° in width. A defect in the internal anal sphincter was diagnosed by the presence of a hyperechoic area, sometimes accompanied by thickening of the damaged ends of the muscle resulting from retraction.

Women were counselled and offered a subsequent vaginal birth, unless they were symptomatic or had abnormal EAUS, and these women were advised to consider an elective caesarean section. However, all women were supported in their decision on birth mode should it differ [11]. All women were routinely offered a 6-month postnatal EAUS clinic appointment to repeat the scans to assess anal sphincter muscle integrity following the subsequent birth. Women recruited to the study were also asked to complete the validated Manchester Health Questionnaire (MHQ) [13] at both the 34-week antenatal clinic appointment and again at the 6-month postnatal clinic appointment. For women who declined to attend the 6-month postnatal EAUS appointment, the MHQ questionnaire was sent by post.

The MHQs were anonymised by using only study participation reference number and, following completion, the participant then sealed them in an envelope and returned them by hand or post. All data required for the study were collected on study-specific MHQ and data collection forms.

The study was designed, undertaken and reported using the Strengthening the Reporting of Observational Studies in Epidemiology (STROBE) statement and checklist [14].

The outcome measure was bowel function and related QoL evaluated through completion of the MHQ at 34 weeks’ gestation and at 6 months postnatally. This validated questionnaire captures bowel function/symptoms experienced within the 4 weeks prior to completion of the questionnaire (faecal urgency, difficulty wiping, poor control of flatus, faecal incontinence) and the consequent impact on QoL reflected in nine QoL domains: general health perception (GHP), incontinence impact (II), role limitations (RL), physical limitations (PL), social limitations (SL), personal relationships (PR), emotions (E), sleep/energy (SE) and Severity Measure (SM). All of the QoL domains have more than one question to assess them and each domain is scored, whereby a lower score equates to less impact on QoL. The MHQ questions concerning bowel function are a symptom index and do not form part of the QoL score, but act as a guide to symptomatology.

For univariate analysis, change in the frequency of each bowel symptom and each of the QoL domains total scores recorded in the postnatal MHQ relative to the antenatal MHQ were categorised as “worsening”, “no change” or “improved”. For multivariate analysis we investigated whether the effect of a subsequent birth on bowel function-related QoL was “none or poor”, and dichotomised postnatal bowel symptoms as either “absent” or “present” (Supplementary Table 1).

Permission was obtained from NRES Committee West Midlands -South Birmingham Local Research Ethics Committee (13/WM/0367). This study was funded by NIHR Clinical Doctoral Research Fellowship—CDRF-2012-03-064.

If bowel symptoms occurred in 20% of women in this study (35 out of 175), the sample size of 175 women would allow a binomial exact 95% confidence interval spanning from 14.3% to 26.7% to be constructed, giving reasonable precision.

Data were analysed using STATA® [15] and SPSS® [16]. Differences in baseline characteristics were analysed using a two-sample *t* test for continuous characteristics, Mann–Whitney *U* test for skewed data, Chi-squared test for categorical characteristics when the numbers in each cell were greater than or equal to 5 and a Fisher’s exact test for categorical characteristics when the numbers in the cell were less than or equal to 5. A *p* value < 0.05 was considered statistically significant.

A multivariate logistic regression model providing odds ratios (OR) and 95% confidence intervals (95% CI), was used to evaluate associations between possible independent characteristics (OASI birth mode, mode of subsequent birth, vaginal interval birth, bowel function following subsequent birth, maternal age at OASI, years between OASI and questionnaire completion, total parity, OASI classification, repair method and birthweight) and the outcome of a poor score (MHQ domain score of ≥ 1) for each of the 9 MHQ QoL domains separately. For the QoL domains of “physical limitations”, “social limitations” and “personal relationships” the bowel symptom of faecal urgency was removed as a contributory characteristic owing to the low number of events.

## Results

**Fig. 1:**
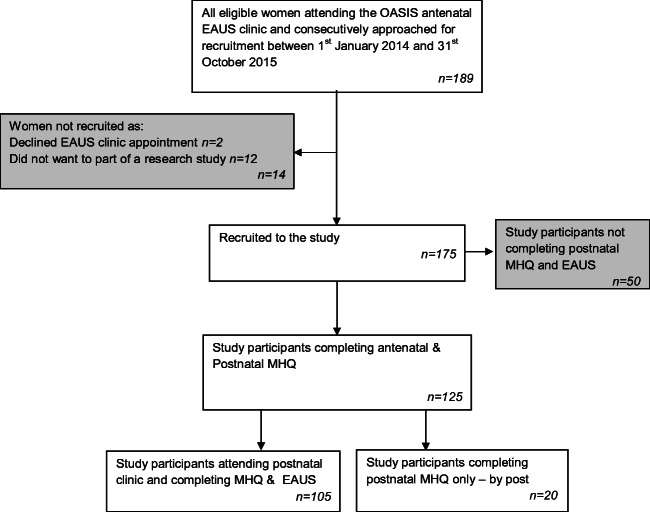
Flow chart to show cohort recruitment. *OASIS* obstetric anal sphincter injuries, *EAUS* endoanal ultrasound, *MHQ* Manchester Health Questionnaire

All 189 eligible women with a previous OASI attending the specialist antenatal clinic for EAUS and to discuss the mode of birth for their current pregnancy were approached for recruitment and 175 took part (92.6%). No women had undergone any further surgery or treatment following their OASI other than routine postnatal physiotherapy. The study recruitment flowchart is presented in Fig. 1. Of these 175 women, all but 2 (98.9%) completed an antenatal MHQ and underwent antenatal EAUS. Mean gestation at recruitment to the study was 32^+4^ weeks.

Of the 175 study participants, 125 (71.4%) were followed up at 6 months; 105 (84%) completed the postnatal MHQ when they attended the postnatal clinic appointment and had an EAUS, and 20 (16%) declined this appointment and so did not have an EAUS and completed the MHQ by post. The mean time period between the study birth and the completion of the postnatal MHQ was 6.8 months (± 2.17 months).

Table 1 shows the baseline maternal, OASI, labour and neonatal characteristics of all women recruited to the study and compares those who did and did not complete postnatal MHQ. The only significant differences between groups were the method of initial OASI repair, with more unspecified results in the group that was not followed up, and birthweight, with those not followed up having lower birthweight babies. With regard to baseline bowel function at antenatal MHQ completion, the only significant difference was for control of flatus with more women who completed the postnatal MHQ having had poor control of flatus at the time of antenatal MHQ completion (Supplementary Table 2). For baseline QoL domain scores, the only difference was for the domain of “emotion”, with more women who completed the postnatal MHQ having a score that indicates that bowel symptoms had a negative impact on this QoL domain (Supplementary Table 3).

**Table 1:** Baseline characteristics of all participants—6-month postnatal follow-up and no postnatal follow-up

Characteristics	All women	Postnatal MHQ	No postnatal MHQ	*p* value
	*N* = 175	*n* = 125	*n* = 50	
Maternal characteristics				
Age at OASI, years, mean (SD)	27.8 (4.6)	28.1 (4.4)	27.2 (4.9)	0.294
Ethnicity				
White	85 (48.6)	64 (51.2)	21 (42.0)	
Mixed/multiple	2 (1.1)	0	2 (4.0)	
Asian/Asian British	60 (34.3)	40 (32.0)	20 (40.0)	
Black/African/Caribbean/Black British	22 (12.6)	16 (12.8)	6 (12.0)	
Other/not known	6 (3.4)	5 (4.0)	1 (2.0)	
BMI, mean (SD)	26.5 (5.7)	26.1 (5.2)	27.6 (6.8)	0.159
Parity at recruitment				0.497
1	126 (72.0)	87 (69.6)	39 (78.0)	
2	40 (22.9)	30 (24.0)	10 (20.0)	
≥3	9 (5.1)	8 (6.4)	1 (2.0)	
Gestation at recruitment, weeks, median (IQR)*	32 (31,33)	32 (31,33)	32 (31,33)	0.717
OASI characteristics				
OASI classification				0.334
3A	50 (28.6)	37 (29.6)	13 (26.0)	
3B	58 (33.1)	43 (34.4)	15 (30.0)	
3C/4	24 (13.71)	19 (15.2)	5 (10.0)	
Unspecified	43 (24.6)	26 (20.8)	17 (34.0)	
Method of repair				*0.045*
End-to-end	72 (41.1)	55 (44.0)	17 (34.0)	
Overlap	53 (30.3)	41 (32.8)	12 (24.0)	
Unspecified	50 (28.6)	29 (23.2)	21 (42.0)	
Anal sphincter defect on antenatal EAUS^a^				0.054
Present	42 (24.3)	35 (28.2)	7 (14.3)	
Absent	131 (75.7)	89 (71.8)	42 (85.7)	
Labour characteristics for OASI birth				
Mode of OASI birth				0.058
SVD	105 (60.0)	75 (60.0)	30 (60.0)	
Kiwi/ventouse	17 (9.7)	16 (12.8)	1 (2.0)	
Low/unspecified forceps	46 (26.3)	28 (22.4)	18 (36.0)	
Rotational forceps	7 (4.0)	6 (4.8)	1 (2.0)	
Induction of labour	58 (33.1)	43 (34.4)	15 (30.0)	0.576
Epidural	41 (23.4)	31 (24.8)	10 (20.0)	0.498
Maternal position at birth				0.191
Lithotomy	74 (42.3)	52 (41.6)	22 (44.0)	
Supported sitting	72 (41.1)	47 (37.6)	25 (50.0)	
All fours	4 (2.3)	3 (2.4)	1 (2.0)	
Standing	2 (1.1)	2 (1.6)	0	
Lateral	1 (0.6)	1 (0.8)	0	
Kneeling	10 (5.7)	9 (7.2)	1 (2.0)	
McRoberts	5 (2.9)	5 (4.0)	0	
Squatting	1 (0.6)	0	1 (2.0)	
Not known	6 (3.4)	6 (4.8)	0	
Infant characteristics for OASI birth				
Gestational age, weeks, median (IQR)*	39 (39,40)	40 (39,40)	40 (38,40)	0.197
Birth weight, kg, mean (SD)	3.448 (0.595)	3.538 (0.537)	3.224 (0.676)	*0.001*
Head circumference cm, mean (SD)	34.1 (3.4)	34.2 (3.8)	33.9 (1.7)	0.709

*OASI* obstetric anal sphincter injury, *EAUS* endoanal ultrasound scan, *IQR* interquartile range, *MHQ* Manchester Health Questionnaire, *SVD* spontaneous vaginal delivery

*IQR* interquartile range, *SD* standard deviation

*The *t* test was conducted for continuous parameters (with Mann–Whitney *U* test for skewed data)

^a^*N* = 173 (2 women declined antenatal EAUS)

Among the 125 women followed up there was no evidence of differences in any of the baseline maternal, labour or neonatal characteristics among the 105 women who attended the specialist postnatal EAUS clinic and the 20 who responded by post, except for a significant difference in the presence of anal sphincter defects, with slightly more women with a known anal sphincter defect attending for the postnatal clinic appointment. Bowel function and QoL were also similar in those who attended their appointment and those who completed the MHQ by post (Supplementary Table 4).

Of the 175 antenatal recruits to the study, 60.6% (106 out of 175) had chosen to pursue a vaginal birth and 36.6% (64 out of 175) had opted for an elective caesarean section. The remaining 2.8% (5 out of 175) had made a birth plan that encompassed either a vaginal birth or a caesarean section depending on ante−/intra-partum events. Table 2 shows mode of birth choice in relation to baseline antenatal EAUS findings. Despite an anal sphincter defect diagnosed at the antenatal EAUS, 2 women (1.9%) chose to pursue a vaginal birth. Of the 64 women who chose an elective caesarean section, 62.5% (40 out of 64) had a known anal sphincter defect. Two women (3.1%) chose an elective caesarean section without having an EAUS to determine anal sphincter integrity. Of the 175 women in this study, 105 had a subsequent vaginal birth and 70 had a caesarean section—baseline maternal and neonatal characteristics are shown in Table 3. Women undergoing a caesarean section for their subsequent birth had a slightly higher BMI (*p* = 0.031), and a much higher proportion of women undergoing a subsequent vaginal birth had had a vaginal birth since sustaining an OASI (*p* = <0.001). As to be expected, a much higher proportion of women undergoing a caesarean section for the subsequent birth had bowel symptoms (*p* = <0.001) and anal sphincter defects detected during antenatal EAUS (*p* = <0.001).

**Table 2:** Chosen mode of subsequent birth by the presence of any anal defect (external anal sphincter [EAS] and/or internal anal sphincter [IAS]), number (%)

	Chosen mode of subsequent birth			Total
Anal sphincter defects on EAUS	Vaginal birth	Elective caesarean section	Either	
Any anal sphincter defect present (EAS and/or IAS)	2 (1.9)	40 (62.5)	0	42 (24.0)
No anal sphincter defect	104 (98.1)	22 (34.4)	5 (100)	131 (74.9)
EAUS declined	0	2 (3.1)	0	2 (3.1)
Total	106	64	5	175

*EAUS* endoanal ultrasound

**Table 3:** Baseline maternal characteristics—subsequent birth mode vaginal birth or caesarean section

Characteristics, *n*, (%)	Vaginal birth	Caesarean section	*p* value
	*n* = 105 (60.0)	*n* = 70 (40.0)	
Maternal characteristics			
Age at OASI birth, years, mean (SD)	27.7 (4.7)	28.0 (4.4)	0.653
Time between OASI birth and antenatal questionnaire completion, years, mean (SD)	4.29 (3.39)	3.94 (3.94)	0.460
Ethnicity			0.260
White	47 (44.8)	38 (54.3)	
Mixed/multiple	1 (0.9)	1 (1.4)	
Asian/Asian British	35 (33.3)	25 (35.7)	
Black/African/Caribbean/Black British	17 (16.2)	5 (7.2)	
Other/not known	5 (4.8)	1 (1.4)	
BMI, mean (SD)	25.8 (5.3)	27.8 (6.0)	*0.031*
Parity at recruitment			0.469
1	73 (69.5)	53 (75.7)	
2	25 (23.8)	15 (21.4)	
≥3	7 (6.7)	2 (2.9)	
Vaginal interval birth	26 (24.8)	3 (4.3)	*<0.001*
OASI birth characteristics			
OASI classification			0.188
3A	35 (33.3)	15 (21.4)	
3B	32 (30.5)	26 (37.1)	
3C/4	11 (10.5)	13 (18.6)	
Unspecified	27 (25.7)	16 (22.9)	
Method of repair			0.433
End-to-end	46 (43.8)	26 (37.1)	
Overlap	28 (26.7)	25 (35.7)	
Unspecified	31 (29.5)	19 (27.2)	
Mode of OASI birth			0.131
SVD	69 (65.7)	36 (51.4)	
Kiwi/ventouse	11 (10.5)	6 (8.6)	
Low/unspecified forceps	21 (20.0)	25 (35.7)	
Rotational forceps	4 (3.8)	3 (4.3)	
Neonatal characteristics for OASI birth			
Gestational age, (weeks), median (IQR)	40 (39, 40)	40 (39,40)	0.529
Birth weight, (kg), mean (SD)	3.404 (0.586)	3.513 (0.606)	0.237
Head circumference (cm), mean (SD)	33.8 (4.2)	34.5 (1.7)	0.214
Subsequent birth characteristics			
Maternal request for mode of birth	91 (86.7)	63 (90.0)	0.506
Bowel symptoms	1 (0.9)	19 (27.1)	*<0.001*
Requested mode of study birth achieved^a^	102 (97.1)	63 (90.0)	0.091
Anal sphincter defect on antenatal EAUS	3 (2.9)	38 (54.3)	*<0.001*

*IQR* interquartile range, *OASI* obstetric anal sphincter injury, *SVD* spontaneous vaginal delivery

^a^Excludes the 5 women who were pursuing either a vaginal or caesarean section depending on clinical events

Most of the women, 94.1% (160 out of 170), ended up having their planned mode of birth. Of the 10 women who did not, 4 subsequently changed their mind, 2 who originally booked for elective caesarean section were admitted in established labour and progressed to vaginal birth too quickly for an emergency caesarean section and 4 women had clinical indications necessitating emergency caesarean section. With regard to actual mode of birth in relation to initial OASI classification, more women with a 3a (33.3% vs 21.4%) or unspecified OASI (25.7% vs 22.9%) had a vaginal birth than a caesarean section. However, for both 3b (30.5% vs 37.1%) or 3c/4 (10.5% vs 18.6%) OASI classifications, a higher number of women had a caesarean section than a vaginal birth (Supplementary Table 5). Repeat OASI rate was 3.8% (4 out of 105).

For the 125 women followed up, changes in bowel symptoms between antenatal baseline and 6 months follow-up were similar in women who had a vaginal birth compared with caesarean section; QoL scores were also similar (Table 4).

**Table 4:** A comparison of changes in bowel symptom frequency and Manchester Health Questionnaire (MHQ) quality-of-life (QoL) scores pre- and post-subsequent birth, for vaginal birth versus caesarean section, number (%)

	Mode of birth—vaginal (*n* = 74)			Mode of birth—caesarean section (*n* = 51)			
	Postnatal MHQ bowel frequency compared with antenatal MHQ bowel frequency			Postnatal MHQ bowel frequency compared with antenatal MHQ bowel frequency			
	Worsened	No change	Improved	Worsened	No change	Improved	*p* value
Bowel function following study birth							
Bowel urgency	20 (27.0)	34 (46.0)	20 (27.0)	8 (15.7)	24 (47.1)	19 (37.2)	0.265
Poor control of flatus	15 (20.3)	41 (55.4)	18 (24.3)	8 (15.7)	29 (56.9)	14 (27.4)	0.800
Difficulty with wiping clean	2 (2.7)	69 (93.2)	3 (4.1)	1 (2.0)	47 (92.2)	3 (5.8)	0.865
Faecal leakage—any	9 (12.2)	58 (78.4)	7 (9.4)	4 (7.8)	38 (74.5)	9 (17.7)	0.345
MHQ QoL domain							
General health perception	9 (12.2)	43 (58.1)	22 (29.7)	5 (9.8)	29 (56.9)	17 (33.3)	0.897
Incontinence impact	14 (18.9)	47 (63.5)	13 (17.6)	5 (9.8)	32 (62.8)	14 (27.5)	0.235
Role limitations	11 (14.9)	57 (77.0)	6 (8.1)	6 (11.8)	39 (76.5)	6 (11.8)	0.699
Physical limitations	2 (2.7)	63 (85.1)	9 (12.2)	6 (11.8)	36 (70.6)	9 (17.7)	0.077
Social limitations	4 (5.4)	64 (86.5)	6 (8.1)	3 (5.9)	43 (84.3)	5 (9.8)	0.927
Personal relationships	5 (6.8)	63 (85.1)	6 (8.1)	2 (3.9)	43 (84.3)	6 (11.8)	0.691
Emotions	11 (14.9)	52 (70.3)	11 (14.9)	3 (5.9)	41 (80.4)	7 (13.7)	0.292
Sleep/energy	2 (2.7)	65 (87.8)	7 (9.5)	3 (5.9)	43 (84.3)	5 (9.8)	0.713
Severity measure	5 (6.8)	55 (74.3)	14 (18.9)	6 (11.8)	33 (64.7)	12 (23.3)	0.421

*MHQ* Manchester Health Questionnaire, *QoL* quality of life

Multivariate analysis to examine QoL at 6 months post-subsequent birth, which adjusted for bowel function, mode of birth, maternal, intrapartum, OASI and neonatal characteristics, showed that the odds of poor QoL for the domains of “incontinence impact” (OR 2.91, 95% CI 1.03–8.21) and “physical limitations” (OR 4.56, 95% CI 1.02–20.45) were significantly higher for women who had undergone a caesarean section, and borderline for “sleep/energy” (OR 4.77, 95% CI 0.9–25.17) and “social limitations” (OR 7.37, 95% CI 0.89–6,118), compared with those who had a subsequent vaginal birth. However, the mode of birth was not found to have a significant positive or negative association with any of the other five MHQ QoL domains (Table 5). With regard to parity, the odds of poor QoL for the domains of “incontinence impact” (OR 5.12, 95% CI 1.10–23.97) and “emotions” (OR 8.49, 95% CI 1.87–38.44) were significantly higher for women who had parity ≥3 compared with women who were in their second pregnancy. Interestingly, with regard to the classification of previous OASIS, the odds of poor QOL were significantly lower for women with a previous 3c or 4th degree OASI for the QoL domain of “incontinence impact” (OR 0.17, 95% CI 0.03–0.98) compared with women who had sustained a 3a OASI (Supplementary Table 6).

**Table 5:** Multivariate analysis of postnatal bowel function, maternal, intrapartum, obstetric anal sphincter injury (OASI) and neonatal characteristics on poor quality-of-life (QoL) scores post-subsequent birth. Results comparing caesarean section with vaginal delivery for each of the QoL domains

MHQ QoL domains	aOR^b^	95% CI	*p*
General health perception	1.42	(0.61–3.30)	0.418
Incontinence impact	2.91	(1.03–8.21)	*0.044*
Role limitations	0.84	(0.28–2.48)	0.749
Physical limitations^a^	4.56	(1.02–20.45)	*0.048*
Social limitations^a^	7.37	(0.89–61.18)	0.064
Personal relationships^a^	0.90	(0.14–5.61)	0.908
Emotions	2.20	(0.79–6.11)	0.131
Sleep/energy	4.77	(0.90–25.17)	0.066
Severity measures	2.18	(0.69–6.82)	0.182

*MHQ* Manchester Health Questionnaire, *QoL* quality of life

^**a**^Bowel symptom of faecal urgency was removed as a contributory characteristic owing to the low number of events) at initial hospital review, maternal age at OASI, years between OASI and questionnaire completion, total parity, OASI classification, repair method and birthweight

^b^OR adjusted for OASI birth mode, mode of study birth, vaginal interval birth, bowel symptoms

Of the 105 women who had postnatal EAUS, 77 had no anal sphincter defect and 28 did. In each of these subgroups there was no evidence of association between mode of birth and change in frequency of bowel symptoms. However, for the 77 women who had no anal sphincter defect, women who had a caesarean section were more likely to have an improved “physical limitations” QOL domain score (*p =* 0.014; Supplementary Tables 7 and 8).

## Discussion

This high-quality cohort study designed specifically to assess the impact of the mode of subsequent birth on bowel function and related quality of life in pregnant women who had previously sustained OASI counselled in line with RCOG Green-top recommendations. In this sample, there was no significant association between whether the birth was vaginal or caesarean section and worsening, no change or improvement in any bowel symptoms or individual MHQ QoL domain scores. However, multivariate analysis showed that, despite there being a higher proportion of women having a subsequent caesarean section reporting an improvement in bowel symptoms and a higher proportion of women undergoing a subsequent vaginal birth experiencing a worsening of bowel symptoms, the odds of poor QoL for the domains of “incontinence impact” and “physical limitations” were significantly higher for women undergoing a subsequent caesarean section.

This present cohort study of consecutive women was also designed to investigate the impact of a subsequent birth by any mode on bowel function-related QoL for women with a previous OASI using recommendations based on current RCOG guidelines. Others have been audits of service provision with consequent biases. This study satisfies all of the STROBE checklist requirements, no other similar published studies having reported compliance with this checklist [4–9]. Consequently, this study is of high methodological quality, which limits bias and satisfies the research recommendations highlighted in the published systematic review [3].

Repeat OASI rate in this study was 3.8%, lower than that found in other studies [17]. Also, for women in this study, the only significant differences in baseline characteristics between those undergoing a subsequent vaginal birth or a caesarean section were BMI and having already had a vaginal birth since the birth during which an OASI was sustained. This contrasts with findings by D’Souza et al. who found ethnicity, age, OASI classification, previous birthweight and mode of OASI birth to be significant factors among women undergoing a subsequent vaginal birth or elective caesarean section [17]. However, for women in this study, all EAUS and pre-birth counselling regarding mode of study birth was undertaken by one single clinician (specialist midwife—perineal trauma and author of this paper). This not only reduces inter-rater variability that other studies have not acknowledged [4–9], it may also explain the differences in findings between this study and that of D’Souza et al. [17], as women receive specialist counselling that allows discussion of all relevant factors, including sensitive information that some cultures may feel reluctant to divulge, in order to facilitate informed decision-making.

This study also reports on the impact of the subsequent birth mode, regardless of whether it was planned or against advice, thus reducing bias and providing evidence for women prior to the birth of the possible consequences of a birth mode that is not as planned or against recommendations [4, 5, 7]. Clinical experience confirms that for women with a previous OASI, their main concern when deciding on mode of subsequent birth is the possibility of deterioration in quality of life through a worsening of bowel function. Therefore, a further strength is the decision to report on a change in incidence as the measure of the impact of the subsequent birth on bowel function. This provides a more meaningful reflection of impact and assistance for future women with decision-making.

To our knowledge, this is the first study to have undertaken multivariate analysis to evaluate the interaction between possible independent characteristics, including mode of subsequent birth, with the outcome of poor QoL.

Our results show no significant association between the mode of subsequent birth and a change in bowel symptom frequency for any of the bowel symptoms, similar to findings from other studies [5, 8, 9]. Interestingly, for two bowel symptoms a greater proportion of women had an improvement in bowel symptoms at 6 months postnatally. Scheer et al. [4] found bowel function improvements in women undergoing subsequent vaginal and caesarean birth. Such improvements could be due to physiological changes that occur during the postnatal period, when hormones and consequent bowel physiology are beginning to return to a pre-pregnancy state. This contrasts with findings from Reid et al. [18], who found that more women with a previous OASI who had a subsequent birth by caesarean section had anal incontinence at a 3-year follow-up than those who had a subsequent vaginal birth (*p* = 0.012). They proposed that this was because a caesarean section was recommended to symptomatic women and an increase in the proportion of these women was attributed to a worsening of pre-existing symptoms. However, the same recommendations were used for women in this study, and therefore improvement in bowel symptoms may not simply be due to elimination of the risk of further somatic trauma to the sphincter complex, pelvic floor and innervation but may also be influenced by achieving the desired mode of birth, learning to cope with bowel symptoms or actual improvement because of management interventions such as dietary changes or physiotherapy [6].

Despite QoL being an important indicator for women with a previous OASI when deciding on future pregnancy and birth mode, research into this area is limited, with only one small study having investigated the association between mode of the subsequent birth and QoL through univariate analysis. In this small study (*n* = 44), Sheer et al. found a significant negative impact on QoL for symptomatic women recommended to undergo caesarean section versus a vaginal birth [4]. This contradicts with our univariate findings that did not demonstrate a significant association between whether the subsequent birth was vaginal or caesarean section and worsening, no change or improvement in QoL scores. However, multivariate analysis undertaken in our study showed a poor QoL for two MHQ domains for women undergoing subsequent caesarean section. Therefore, it may be that the poor QoL for the women undergoing a recommended caesarean section could be attributed to a continuation of bowel symptoms that were present prior to the birth and a reason why the mode of caesarean was chosen, not necessarily a worsening after the birth.

Follow-up response was relatively high, at 71.4%, but as with all cohort studies that include a follow-up assessment, possible effects of even relatively low attrition must be considered. Baseline data were available for all women, and comparisons of the baseline characteristics for responders and non-responders with the postnatal MHQ, women who completed the postnatal MHQ by attending the hospital clinic appointment and those who completed the MHQ by post, and women who underwent a subsequent vaginal birth and those who had a caesarean section showed few differences.

Another limitation to this study is that the results of some of the multivariate analyses must be reviewed with caution as the confidence intervals were large; therefore, the precision of the estimates is low.

The findings from this study have important implications for clinicians caring for women with a previous OASI considering a subsequent pregnancy. This study provides robust observational evidence not previously available to support the use of current RCOG guidelines for mode of subsequent birth for women with a previous OASI. A vaginal birth is suitable for women with no bowel symptoms and normal EAUS, although in some women, worsening of bowel symptoms may occur, but this did not have a negative impact on QoL. A caesarean section remains a reasonable mode of birth for women who have abnormal bowel function *and/or* abnormal EAUS findings; however, QoL may be poorer in some domains, possibly through continuation, but not necessarily worsening, of symptoms.

Further research is needed to investigate the most suitable mode of subsequent birth for women who choose to pursue or end up undergoing a mode of birth that is contrary to current RCOG guidance. Regardless of how the research is undertaken (either as a randomised controlled trial or as a cohort study), to ensure timely conclusion and reach the necessary power to address important outcomes, it is likely that such research will need to be multi-centre/international.

## Electronic supplementary material

ESM 1 (DOCX 15 kb)

ESM 2 (DOCX 18 kb)

ESM 3 (DOCX 18 kb)

ESM 4 (DOCX 28 kb)

ESM 5 (DOCX 15 kb)

ESM 6 (DOCX 67 kb)

ESM 7 (DOCX 16 kb)

ESM 8 (DOCX 18 kb)
