# Supplementary material for: The impact of mode of subsequent birth after obstetric anal sphincter injury on bowel function and related quality of life: a cohort study
Source: Int Urogynecol J. 2020 Feb 24;31(11):2237–45. doi: 10.1007/s00192-020-04234-3 (PMC7561530; doi:10.1007/s00192-020-04234-3)
Supplement: Supplementary file 1 — (DOCX 15 kb) [file 192_2020_4234_MOESM1_ESM.docx]

**Supplementary Table 1**

| Bowel function/symptoms | |
| --- | --- |
| Description | Variable classification |
| Frequency of bowel function/symptom as recorded in the MHQ. | ‘Never’  ‘Occasionally’  ‘Sometimes’  ‘Most of the time’  ‘All of the time’ |
| Any change in the frequency of the bowel symptom recorded in the postnatal MHQ compared to that recorded in the antenatal MHQ. | ‘Worsened’ - when the frequency of the symptom at postnatal MHQ completion was recorded as having occurred more often than that recorded in the antenatal MHQ.  ‘No change’ - when the frequency of the bowel symptom at postnatal MHQ completion was the same than that recorded in the antenatal MHQ.  ‘Improved’ - when the frequency of the bowel symptom at postnatal MHQ completion was less than that recorded in the antenatal MHQ. |
| The presence of a bowel symptom | ‘Absent’ - when the frequency of the symptom was recorded as ‘Never’ on the MHQ.  ‘Present’ when the frequency of the symptom was recorded as ‘Occasionally’ or ‘Sometimes’ or ‘Most of the time’ or ‘All of the time’ on the MHQ. |
| The presence of any type of faecal leakage with a recording of any of the following symptoms at any frequency - passive leakage, leakage with coughing, leaking with walking, any loose or solid leakage or leaking with sexual intercourse. | ‘Any faecal leakage ‘ |
| QoL | |
| Description | Variable classification |
| QoL domain total score calculated from a scoring system whereby a lower score equates to less impact on QoL | 0  1-25  26-50  51-75  76-100 |
| Any change in the QoL domain score in the postnatal MHQ compared to the corresponding QoL domain score in the antenatal MHQ. | ‘Worsened’ - when the QoL domain score at postnatal MHQ completion was higher than the corresponding domain score in the antenatal MHQ.  ‘No change’ - when the QoL domain score at postnatal MHQ completion was the same as the corresponding domain score in the antenatal MHQ.  ‘Improved’ - when the QoL domain score at postnatal MHQ completion was lower than the corresponding domain score in the antenatal MHQ. |
| The effect on QoL | ‘None’ - A score of 0 was deemed indicative of no effect on QoL as this score is calculated from the answers of ‘never’  ‘Poor’ - A score of ≥ 1 was deemed indicative of a negative effect on QoL as this score is calculated from the answers of ‘rarely’, ‘sometimes’, ‘often’ and ‘always’ |
