# Supplementary material for: The impact of mode of subsequent birth after obstetric anal sphincter injury on bowel function and related quality of life: a cohort study
Source: Int Urogynecol J. 2020 Feb 24;31(11):2237–45. doi: 10.1007/s00192-020-04234-3 (PMC7561530; doi:10.1007/s00192-020-04234-3)
Supplement: Supplementary file 2 — (DOCX 18 kb) [file 192_2020_4234_MOESM2_ESM.docx]

**Supplementary table 2** Baseline characteristics of participants – six month postnatal MHQ and no postnatal MHQ

| Characteristics | All women | Postnatal MHQ | No postnatal MHQ | *p*-value |
| --- | --- | --- | --- | --- |
|  | N=175 | N=125 | N=50 |  |
| **Bowel function at antenatal questionnaire completion** |  |  |  |  |
| Bowel urgency |  |  |  | 0.428 |
| Never | 44 (25.1) | 28 (22.4) | 16 (32.0) |  |
| Occasionally | 69 (39.4) | 50 (40.0) | 19 (38.0) |  |
| Sometimes | 50 (28.6) | 39 (31.2) | 11 (22.0) |  |
| Most of the time | 10 (5.7) | 6 (4.8) | 4 (8.0) |  |
| All of the time | 2 (1.1) | 2 (1.6) | 0 |  |
| Difficulty wiping clean |  |  |  | 0.219 |
| Never | 110 (62.9) | 73 (58.4) | 37 (74.0) |  |
| Occasionally | 35 (20.0) | 30 (24.0) | 5 (10.0) |  |
| Sometimes | 15 (8.6) | 11 (8.8) | 4 (8.0) |  |
| Most of the time | 13 (7.4) | 9 (7.2) | 4 (8.0) |  |
| All of the time | 2 (1.1) | 2 (1.6) | 0 |  |
| Poor control of flatus |  |  |  | *0.001* |
| Never | 91 (52.0) | 53 (42.4) | 38 (76.0) |  |
| Occasionally | 45 (25.7) | 38 (30.4) | 7 (14.0) |  |
| Sometimes | 20 (11.4) | 18 (14.4) | 2 (4.0) |  |
| Most of the time | 15 (8.6) | 14 (11.2) | 1 (2.0) |  |
| All of the time | 4 (2.3) | 2 (1.6) | 2 (4.0) |  |
| Faecal leakage- passive only |  |  |  | 0.353 |
| Never | 167 (95.4) | 118 (94.4) | 49 (98.0) |  |
| Occasionally | 5 (2.9) | 5 (4.0) | 0 |  |
| Sometimes | 3 (1.7) | 2 (1.6) | 1 (2.0) |  |
| Most of the time | 0 | 0 | 0 |  |
| All of the time | 0 | 0 | 0 |  |
| Faecal leakage with coughing/sneezing |  |  |  | 0.501 |
| Never | 150 (85.7) | 107 (85.6) | 43 (86.0) |  |
| Occasionally | 17 (9.7) | 13 (10.4) | 4 (8.0) |  |
| Sometimes | 6 (3.4) | 3 (2.4) | 3 (6.0) |  |
| Most of the time | 0 | 2 (1.6) | 0 |  |
| All of the time | 0 | 0 | 0 |  |
| Faecal leakage with walking |  |  |  | 0.433 |
| Never | 167 (95.4) | 118 (94.4) | 49 (98.0) |  |
| Occasionally | 4 (2.3) | 4 (3.2) | 0 |  |
| Sometimes | 4 (2.3) | 3 (2.4) | 1 (2.0) |  |
| Most of the time | 0 | 0 | 0 |  |
| All of the time | 0 | 0 | 0 |  |
| Faecal leakage during SI |  |  |  | 0.225 |
| Never | 172 (95.4) | 124 (99.2) | 48 (96.0) |  |
| Occasionally | 4 (2.3) | 1 (0.8) | 1 (2.0) |  |
| Sometimes | 4 (2.3) | 0 | 1 (2.0) |  |
| Most of the time | 0 | 0 | 0 |  |
| All of the time | 0 | 0 | 0 |  |
| Faecal leakage – loose stool |  |  |  | 0.442 |
| Never | 141 (98.3) | 99 (79.2) | 42 (84.0) |  |
| Occasionally | 2 (1.1) | 15 (12.0) | 4 (8.0) |  |
| Sometimes | 1 (0.6) | 9 (7.2) | 2 (4.0) |  |
| Most of the time | 0 | 1 (0.8) | 2 (4.0) |  |
| All of the time | 0 | 1 (0.8) | 0 |  |
| Faecal leakage – solid stool |  |  |  |  |
| Never | 175 (100.0) | 125 (100.0) | 50 (100.0) | ----- |
| Any faecal leakage |  |  |  | 0.956 |
| Yes | 130 (74.3) | 32 (25.6) | 13 (26.0) |  |
| No | 45 (25.7) | 93 (74.4) | 37 (74.0) |  |

IQR: interquartile range; SD: standard deviation.

The *t* test was conducted for continuous parameters (with Mann-Whitney *U* test for skewed data) ^¥^, and *χ^2^* test for categorical variables with missing excluded as appropriate due to small numbers^≠^
