# Supplementary material for: The impact of mode of subsequent birth after obstetric anal sphincter injury on bowel function and related quality of life: a cohort study
Source: Int Urogynecol J. 2020 Feb 24;31(11):2237–45. doi: 10.1007/s00192-020-04234-3 (PMC7561530; doi:10.1007/s00192-020-04234-3)
Supplement: Supplementary file 3 — (DOCX 18 kb) [file 192_2020_4234_MOESM3_ESM.docx]

**Supplementary table 3** Baseline characteristics of participants QoL – six month postnatal MHQ and no postnatal MHQ

| Characteristics | All women | Postnatal MHQ | No postnatal MHQ | *p*-value |
| --- | --- | --- | --- | --- |
|  | N=175 | N=125 | N=50 |  |
| **QoL domain scores at antenatal questionnaire completion** |  |  |  |  |
| General Health Perception (GHP) |  |  |  | 0.628 |
| 0 | 72 (41.1) | 51 (40.8) | 21 (42.0) |  |
| 1-25 | 81 (46.3) | 56 (44.8) | 25 (50.0) |  |
| 26-50 | 20 (11.4) | 16 (12.8) | 4 (8.0) |  |
| 51-75 | 2 (1.1) | 2 (1.6) | 0 |  |
| 76-100 | 0 | 0 | 0 |  |
| Incontinence Impact (II) |  |  |  | 0.076 |
| 0 | 101 (57.7) | 67 (53.6) | 34 (68.0) |  |
| 1-25 | 49 (28.0) | 35 (28.0) | 14 (28.0) |  |
| 26-50 | 15 (8.6) | 14 (11.2) | 1 (2.0) |  |
| 51-75 | 8 (4.6) | 8 (6.4) | 0 |  |
| 76-100 | 2 (1.1) | 1 (0.8) | 1 (2.0) |  |
| Role Limitations (RL) |  |  |  | 0.808 |
| 0 | 47 (26.9) | 32 (25.6) | 15 (30.0) |  |
| 1-25 | 117 (66.9) | 85 (68.0) | 32 (64.0) |  |
| 26-50 | 9 (5.1) | 7 (5.6) | 2 (4.0) |  |
| 51-75 | 2 (1.1) | 1 (0.8) | 1 (2.0) |  |
| 76-100 | 0 | 0 | 0 |  |
| Physical Limitations (PL) |  |  |  | 0.090 |
| 0 | 145 (82.9) | 99 (79.2) | 46 (92.0) |  |
| 1-25 | 16 (9.1) | 14 (11.2) | 2 (4.0) |  |
| 26-50 | 12 (6.9) | 11 (8.8) | 1 (2.0) |  |
| 51-75 | 1 (0.6) | 0 | 1 (2.0) |  |
| 76-100 | 1 (0.6) | 1 (0.8) | 0 |  |
| Social Limitations (SL) |  |  |  | 0.226 |
| 0 | 153 (87.4) | 106 (84.8) | 47 (94.0) |  |
| 1-25 | 17 (9.7) | 15 (12.0) | 2 (4.0) |  |
| 26-50 | 3 (1.7) | 3 (2.4) | 0 |  |
| 51-75 | 0 | 1 (0.8) | 1 (2.0) |  |
| 76-100 | 2 (1.1) | 0 | 0 |  |
| Personal Relationships (PR) |  |  |  | 0.053 |
| 0 | 158 (87.4) | 109 (87.2) | 49 (98.0) |  |
| 1-25 | 17 (9.7) | 14 (11.2) | 0 |  |
| 26-50 | 3 (1.7) | 1 (0.8) | 0 |  |
| 51-75 | 0 | 0 | 1 (2.0) |  |
| 76-100 | 2 (1.1) | 1 (0.8) | 0 |  |
| Emotions (E) |  |  |  | *0.012* |
| 0 | 125 (71.4) | 80 (64.0) | 45 (90.0) |  |
| 1-25 | 28 (16.0) | 25 (20.0) | 3 (6.0) |  |
| 26-50 | 15 (8.6) | 14 (11.2) | 1 (2.0) |  |
| 51-75 | 4 (2.3) | 4 (3.2) | 0 |  |
| 76-100 | 3 (1.7) | 2 (1.6) | 1 (2.0) |  |
| Sleep/Energy (SE) |  |  |  | 0.443 |
| 0 | 150 (85.7) | 105 (84.0) | 45 (90.0) |  |
| 1-25 | 16 (9.1) | 12 (9.6) | 4 (8.0) |  |
| 26-50 | 6 (3.4) | 6 (4.8) | 0 |  |
| 51-75 | 3 (1.7) | 2 (1.6) | 1 (2.0) |  |
| 76-100 | 0 | 0 | 0 |  |
| Severity Measure (SM) |  |  |  | 0.155 |
| 0 | 119 (68.0) | 79 (63.2) | 40 (80.0) |  |
| 1-25 | 40 (22.9) | 32 (25.6) | 8 (16.0) |  |
| 26-50 | 10 (5.7) | 9 (7.2) | 1 (2.0) |  |
| 51-75 | 4 (2.3) | 4 (3.2) | 0 |  |
| 76-100 | 2 (1.1) | 1 (0.8) | 1 (2.0) |  |

IQR: interquartile range; SD: standard deviation.

The *t* test was conducted for continuous parameters (with Mann-Whitney *U* test for skewed data) ^¥^, and *χ^2^* test for categorical variables with missing excluded as appropriate due to small numbers^≠^
