# Supplementary material for: The impact of mode of subsequent birth after obstetric anal sphincter injury on bowel function and related quality of life: a cohort study
Source: Int Urogynecol J. 2020 Feb 24;31(11):2237–45. doi: 10.1007/s00192-020-04234-3 (PMC7561530; doi:10.1007/s00192-020-04234-3)
Supplement: Supplementary file 4 — (DOCX 28 kb) [file 192_2020_4234_MOESM4_ESM.docx]

| **Supplementary Table 4** Baseline characteristics of participants –postnatal clinic follow-up and postnatal postal follow-up | | | | |  |
| --- | --- | --- | --- | --- | --- |
|  |  | | | |  |
| Characteristics | Postnatal clinic follow-up | Postnatal postal follow-up |  | *p*-value |  |
|  | N=105 | N=20 |  |  |  |
| **Maternal characteristics** |  |  |  |  |  |
| Age at OASIS (years), mean [SD] | 27.9 [4.4] | 28.7 [4.5] |  | 0.494 |  |
| Ethnicity |  |  |  | 0.473 |  |
| White | 52 (49.5) | 12 (60.0) |  |  |  |
| Mixed/Multiple | 0 | 10 (0.0) |  |  |  |
| Asian/Asian British | 33 (31.4) | 7 (35.0) |  |  |  |
| Black/African/Caribbean/Black British | 15 (14.3) | 1 (5.0) |  |  |  |
| Other/Not Known | 5 (4.8) | 0 |  |  |  |
| BMI, mean [SD] | 26.2 [5.3] | 26.0 [4.5] |  | 0.913 |  |
| Parity at recruitment |  |  |  | 0.472 |  |
| 1 | 75 (71.4) | 12 (60.0) |  |  |  |
| 2 | 23 (21.9) | 7(35.0) |  |  |  |
| ≥3 | 7 (6.7) | 1 (5.0) |  |  |  |
| **OASIS characteristics** |  |  |  |  |  |
| OASIS classification |  |  |  | 0.918 |  |
| 3A | 31 (29.5) | 6 (30.0) |  |  |  |
| 3B | 37 (35.2) | 6 (30.0) |  |  |  |
| 3C/4 | 15 (14.3) | 4 (20.0) |  |  |  |
| Unspecified | 22 (21.0) | 4 (20.0) |  |  |  |
| Method of repair |  |  |  | 0.930 |  |
| End-to-end | 46 (43.8) | 9 (45.0) |  |  |  |
| Overlap | 34 (32.4) | 7 (35.0) |  |  |  |
| Unspecified | 25 (23.8) | 4 (20.0) |  |  |  |
| Anal sphincter defect on antenatal EAUS |  |  |  |  |  |
| Present | 30 (28.6) | 5 (26.3) |  | *0.040* |  |
| Absent | 75 (71.4) | 14 (73.7) |  |  |  |
| **Labour characteristics for OASIS birth** |  |  |  |  |  |
| Mode of birth |  |  |  | 0.452 |  |
| SVD | 62 (59.0) | 13 (65.0) |  |  |  |
| Kiwi/ventouse | 15 (14.3) | 1 (5.0) |  |  |  |
| Low/unspecified forceps | 24 (22.9) | 4 (20.0) |  |  |  |
| Rotational forceps | 4 (3.8) | 2 (10.0) |  |  |  |
| Induction of labour | 37 (35.2) | 6 (30.0) |  | 0.651 |  |
| Epidural | 25 (23.8) | 6 (30.0) |  | 0.557 |  |
| Maternal position at birth |  |  |  | 0.078 |  |
| Lithotomy | 45 (42.9) | 7 (35.0) |  |  |  |
| Supported sitting | 39 (37.1) | 8 (40.0) |  |  |  |
| All fours | 1 (1.0) | 2 (10.0) |  |  |  |
| Standing | 2 (1.9) | 0 |  |  |  |
| Lateral | 0 | 1 (5.0) |  |  |  |
| Kneeling | 8 (7.6) | 1 (5.0) |  |  |  |
| McRoberts | 5 (4.8) | 0 |  |  |  |
| Squatting | 0 | 0 |  |  |  |
| Not known | 5 (4.8) | 1 (5.0) |  |  |  |
| **Infant characteristics for OASIS birth** |  |  |  |  |  |
| Gestational age, (weeks), median [IQR]^¥^ | 40 [39, 40] | 40 [38, 40] |  | 0.645 |  |
| **Bowel function at antenatal questionnaire completion** | | | | |  |
| Faecal urgency |  |  |  | 0.292 |  |
| Never | 25 (23.8) | 3 (15.0) |  |  |  |
| Occasionally | 43 (41.0) | 7 (35.0) |  |  |  |
| Sometimes | 29 (27.6) | 10 (50.0) |  |  |  |
| Most of the time | 6 (5.7) | 0 |  |  |  |
| All of the time | 2 (1.9) | 0 |  |  |  |
| Difficulty wiping clean |  |  |  | 0.891 |  |
| Never | 60 (57.1) | 13 (65.0) |  |  |  |
| Occasionally | 25 (23.8) | 5 (25.0) |  |  |  |
| Sometimes | 10 (9.5) | 1 (5.0) |  |  |  |
| Most of the time | 8 (7.6) | 1 (5.0) |  |  |  |
| All of the time | 2 (1.9) | 0 |  |  |  |
| Poor control of flatus |  |  |  | 0.421 |  |
| Never | 43 (41.0) | 10 (50.0) |  |  |  |
| Occasionally | 32 (30.5) | 6 (30.0) |  |  |  |
| Sometimes | 14 (13.3) | 4 (20.0) |  |  |  |
| Most of the time | 14 (13.3) | 0 |  |  |  |
| All of the time | 2 (1.9) | 0 |  |  |  |
| Leakage- passive only |  |  |  | 0.494 |  |
| Never | 98 (93.3) | 20 (100.0) |  |  |  |
| Occasionally | 5 (4.8) | 0 |  |  |  |
| Sometimes | 2 (1.9) | 0 |  |  |  |
| Most of the time | 0 | 0 |  |  |  |
| All of the time | 0 | 0 |  |  |  |
| Leakage with coughing/sneezing |  |  |  | 0.369 |  |
| Never | 91 (86.7) | 16 (80.0) |  |  |  |
| Occasionally | 9 (8.6) | 4 (20.0) |  |  |  |
| Sometimes | 3 (2.9) | 0 |  |  |  |
| Most of the time | 2 (1.9) | 0 |  |  |  |
| All of the time | 0 | 0 |  |  |  |
| Leakage with walking |  |  |  | 0.494 |  |
| Never | 98 (93.3) | 20 (100.0) |  |  |  |
| Occasionally | 4 (3.8) | 0 |  |  |  |
| Sometimes | 3 (2.9) | 0 |  |  |  |
| Most of the time | 0 | 0 |  |  |  |
| All of the time | 0 | 0 |  |  |  |
| Leakage during SI |  |  |  | 0.661 |  |
| Never | 104 (99.0) | 20 (100.0) |  |  |  |
| Occasionally | 1 (1.0) | 0 |  |  |  |
| Sometimes | 0 | 0 |  |  |  |
| Most of the time | 0 | 0 |  |  |  |
| All of the time | 0 | 0 |  |  |  |
| Loose leakage |  |  |  | 0.640 |  |
| Never | 84 (80.0) | 15 (75.0) |  |  |  |
| Occasionally | 13 (12.4) | 2 (10.0) |  |  |  |
| Sometimes | 6 (5.7) | 3 (15.0) |  |  |  |
| Most of the time | 1 (1.0) | 0 |  |  |  |
| All of the time | 1 (1.0) | 0 |  |  |  |
| Solid leakage |  |  |  | ---- |  |
| Never | 105 (100.0) | 20 (100.0) |  |  |  |
| Any bowel leakage |  |  |  | 0.623 |  |
| No | 79 (75.2) | 14 (70.0) |  |  |  |
| Yes | 26 (24.8) | 6 (30.0) |  |  |  |
| **QoL domain scores at antenatal questionnaire completion** | | | | |  |
| General Health Perception (GHP) |  |  |  | 0.600 |  |
| 0 | 43 (41.0) | 8 (40.0) |  |  |  |
| 1-25 | 47 (44.8) | 9 (45.0) |  |  |  |
| 26-50 | 14 (13.3) | 2 (10.0) |  |  |  |
| 51-75 | 1 (1.0) | 1 (5.0) |  |  |  |
| 76-100 | 0 | 0 |  |  |  |
| Incontinence Impact (II) |  |  |  | 0.467 |  |
| 0 | 59 (56.2) | 8 (40.0) |  |  |  |
| 1-25 | 26 (24.8) | 9 (45.0) |  |  |  |
| 26-50 | 12 (11.4) | 2 (10.0) |  |  |  |
| 51-75 | 7 (6.7) | 1 (5.0) |  |  |  |
| 76-100 | 1 (1.0) | 0 |  |  |  |
| Role Limitations (RL) |  |  |  | 0.741 |  |
| 0 | 25 (23.8) | 7 (35.0) |  |  |  |
| 1-25 | 73 (69.5) | 12 (60.0) |  |  |  |
| 26-50 | 6 (5.7) | 1 (5.0) |  |  |  |
| 51-75 | 1 (1.0) | 0 |  |  |  |
| 76-100 | 0 | 0 |  |  |  |
| Physical Limitations (PL) |  |  |  | 0.967 |  |
| 0 | 83 (79.0) | 16 (80.0) |  |  |  |
| 1-25 | 12 (11.4) | 2 (10.0) |  |  |  |
| 26-50 | 9 (8.6) | 2 (10.0) |  |  |  |
| 51-75 | 1 (1.0) | 0 |  |  |  |
| 76-100 | 0 | 0 |  |  |  |
| Social Limitations (SL) |  |  |  | 0.553 |  |
| 0 | 90 (85.7) | 16 (80.0) |  |  |  |
| 1-25 | 11 (10.5) | 4 (20.0) |  |  |  |
| 26-50 | 3 (2.9) | 0 |  |  |  |
| 51-75 | 1 (1.0) | 0 |  |  |  |
| 76-100 | 0 | 0 |  |  |  |
| Personal Relationships (PR) |  |  |  | 0.538 |  |
| 0 | 93 (88.6) | 16 (80.0) |  |  |  |
| 1-25 | 10 (9.5) | 4 (20.0) |  |  |  |
| 26-50 | 1 (1.0) | 0 |  |  |  |
| 51-75 | 1 (1.0) | 0 |  |  |  |
| 76-100 | 0 | 0 |  |  |  |
| Emotions (E) |  |  |  | 0.783 |  |
| 0 | 67 (63.8) | 13 (65.0) |  |  |  |
| 1-25 | 20 (19.0) | 5 (25.0) |  |  |  |
| 26-50 | 13 (12.4) | 1 (5.0) |  |  |  |
| 51-75 | 3 (2.9) | 1 (5.0) |  |  |  |
| 76-100 | 3 (1.9) | 0 |  |  |  |
| Sleep/Energy (SE) |  |  |  | 0.304 |  |
| 0 | 89 (84.8) | 16 (80.0) |  |  |  |
| 1-25 | 11 (10.5) | 1 (5.0) |  |  |  |
| 26-50 | 4 (3.8) | 2 (10.0) |  |  |  |
| 51-75 | 1 (1.0) | 1 (5.0) |  |  |  |
| 76-100 | 0 | 0 |  |  |  |
| Severity Measure (SM) |  |  |  | 0.478 |  |
| 0 | 66 (62.9) | 13 (65.0) |  |  |  |
| 1-25 | 25 (23.8) | 7 (35.0) |  |  |  |
| 26-50 | 9 (8.6) | 0 |  |  |  |
| 51-75 | 4 (3.8) | 0 |  |  |  |
| 76-100 | 1 (1.0) | 0 |  |  |  |
| IQR: interquartile range; SD: standard deviation.  The *t* test was conducted for continuous parameters (with Mann-Whitney *U* test for skewed data) ^¥^, and *χ^2^* test for categorical variables with missing excluded as appropriate due to small numbers^≠^ | | | | | |
