# Supplementary material for: The impact of mode of subsequent birth after obstetric anal sphincter injury on bowel function and related quality of life: a cohort study
Source: Int Urogynecol J. 2020 Feb 24;31(11):2237–45. doi: 10.1007/s00192-020-04234-3 (PMC7561530; doi:10.1007/s00192-020-04234-3)
Supplement: Supplementary file 5 — (DOCX 15 kb) [file 192_2020_4234_MOESM5_ESM.docx]

|  | Vaginal birth, n= 105 | | | | Caesarean section, n=70 | | | |  |
| --- | --- | --- | --- | --- | --- | --- | --- | --- | --- |
|  | SVD | Kiwi | Forceps | Total | Elective caesarean section | Emergency caesarean section – prior to labour | Emergency caesarean section – during labour | Total | Total |
| Previous OASI classification, n (%) |  |  |  |  |  |  |  |  |  |
| 3A | 34 (34.3) | 0 | 1(50.0) | 35 | 14 (23.7) | 0 | 1 (14.2) | 15 (21.4) | 50 |
| 3B | 30 (30.3) | 1(25.0) | 1 (50.0) | 32 | 21 (35.6) | 3 (75.0) | 2 (28.6) | 26 (37.1) | 58 |
| 3C/4 | 9 (9.1) | 2 (50.0) | 0 | 11 | 11 (18.6) | 0 | 2 (28.6) | 13 (18.6) | 24 |
| Unspecified | 26 (26.3) | 1 (25.0) | 0 | 27 | 13 (22.0) | 1 (25.0) | 2 (28.6) | 16 (22.9) | 43 |
| Total | 99 (100) | 4 (100) | 2 (100) | 105 (100) | 59 (100) | 4 (100) | 7 (100) | 70 (100) | 175 |

Supplementary Table 5 Actual mode of study birth in relation to the initial OASI classification
