# Supplementary material for: The impact of mode of subsequent birth after obstetric anal sphincter injury on bowel function and related quality of life: a cohort study
Source: Int Urogynecol J. 2020 Feb 24;31(11):2237–45. doi: 10.1007/s00192-020-04234-3 (PMC7561530; doi:10.1007/s00192-020-04234-3)
Supplement: Supplementary file 6 — (DOCX 67 kb) [file 192_2020_4234_MOESM6_ESM.docx]

**Supplementary Table 6**

|  |  | **Postnatal MHQ QoL domains** | | | | | | | | | | | | | | | | | | | | | | | | |  |  |  |  |  |  |  |  |
| --- | --- | --- | --- | --- | --- | --- | --- | --- | --- | --- | --- | --- | --- | --- | --- | --- | --- | --- | --- | --- | --- | --- | --- | --- | --- | --- | --- | --- | --- | --- | --- | --- | --- | --- |
| **Characteristic (n/125)** |  | **General Health Perception** | | | | **Incontinence Impact** | | | | | | | **Role Limitations** | | | | | | | | | | | | | |  |  |  |  |  |  |  |  |
|  |  | OR | 95% CI | *p* | | OR | | 95% CI | | *p* | | | OR | | 95% CI | | | | | | | *p* | | | | | |  |  |  |  |  |  |  |
| **Bowel symptoms at postnatal MHQ completion** | |  |  |  | |  | |  | |  | | |  | | | | | |  | | | | | |  | | | | |  | | |  |  |
| Faecal urgency |  |  |  |  | |  | |  | |  | | |  | | | | | |  | | | | | |  | | | | |  | | |  |  |
| Absent | (28) |  | Reference |  | |  | | Reference | |  | | |  | | | | | | Reference | | | | |  | | | | | |  | | |  |  |
| Present | (97) | 2.38 | (0.80-7.14) | 0.121 | | 0.93 | | (0.26-3.32) | | 0.912 | | | 5.10 | | | | | | (1.32-19.75) | | | | | *0.018* | | | | | |  | | |  |  |
| Difficulty wiping clean |  |  |  |  | |  | |  | |  | | |  | | | | | |  | | | | |  | | | | | |  | | |  |  |
| Absent | (73) |  | Reference |  | |  | | Reference | |  | | |  | | | | | | Reference | | | | |  | | | | | |  | | |  |  |
| Present | (52) | 0.33 | (0.12-0.87) | *0.025* | | 6.44 | | (2.13-19.53) | | *0.001* | | | 0.43 | | | | | | (0.13-1.51) | | | | | 0.189 | | | | | |  | | |  |  |
| Poor control of flatus |  |  |  |  | |  | |  | |  | | |  | | | | | |  | | | | |  | | | | | |  | | |  |  |
| Absent | (53) |  | Reference |  | |  | | Reference | |  | | | Reference | | | | | | | | | | | | | | | | |  | | |  |  |
| Present | (72) | 0.83 | (0.31-2.16) | 0.703 | | 5.07 | | (1.65-15.58) | | *0.005* | | | 1.22 | | | | | | (0.37-4.07) | | | | | 0.743 | | | | | |  | | |  |  |
| Any faecal leakage |  |  |  |  | |  | |  | |  | | |  | | | | | |  | | | | |  | | | | | |  | | |  |  |
| Absent | (93) |  | Reference |  | |  | | Reference | |  | | | Reference | | | | | | | | | | | | | | | | |  | | |  |  |
| Present | (32) | 1.90 | (0.68-5.28) | 0.220 | | 1.83 | | (0.58-5.69) | | 0.301 | | | 3.01 | | | | | | (0.55-16.54) | | | | | 0.206 | | | | | |  | | |  |  |
| **Maternal characteristics** |  |  |  |  | |  | |  | |  | | |  | | | | | |  | | | | |  | | | | | |  | | |  |  |
| Age at OASIS |  | 1.00 | (0.91-1.10) | 0.923 | | 0.91 | | (0.82-1.02) | | 0.107 | | | 1.12 | | | | | | (0.98-1.28) | | | | | 0.094 | | | | | |  | | |  |  |
| Vaginal interval birth(s) |  |  |  |  | |  | |  | |  | | |  | | | | | |  | | | | |  | | | | | |  | | |  |  |
| None | (103) |  | Reference |  | |  | | Reference | |  | | |  | | | | | | Reference | | | | |  | | | | | |  | | |  |  |
| ≥ 1 | (22) | 3.71 | (0.77-17.81) | 0.102 | | 0.40 | | (0.07-2.40) | | 0.316 | | | 15.80 | | | | | | (1.01-247.21) | | | | | *0.049* | | | | | |  | | |  |  |
| Parity (all birth modes) |  |  |  |  | |  | |  | |  | | |  | | | | | |  | | | | |  | | | | | |  | | |  |  |
| 2 | (87) |  | Reference |  | |  | | Reference | |  | | | Reference | | | | | | | | | | | | | | | | |  | | |  |  |
| ≥ 3 | (38) | 0.83 | (0.23-2.90) | 0.765 | | 5.12 | | (1.10-23.97) | | *0.038* | | | 0.81 | | | | | | (1.19-3.49) | | | | | 0.782 | | | | | |  | | |  |  |
| Mode of study birth |  |  |  |  | |  | |  | |  | | |  | | | | | |  | | | | |  | | | | | |  | | |  |  |
| Vaginal | (74) |  | Reference |  | |  | | Reference | |  | | |  | | | | | | Reference | | | | |  | | | | | |  | | |  |  |
| Caesarean section | (51) | 1.42 | (0.61-3.30) | 0.418 | | 2.91 | | (1.03-8.21) | | *0.044* | | | 0.84 | | | | | | (0.28-2.48) | | | | | 0.749 | | | | | |  | | |  |  |
| **Intrapartum characteristics** |  |  |  |  | |  | |  | |  | | |  | | | | | |  | | | | |  | | | | | |  | | |  |  |
| OASIS birth mode |  |  |  |  | |  | |  | |  | | |  | | | | | |  | | | | |  | | | | | |  | | |  |  |
| SVD | (75) |  | Reference |  | |  | | Reference | |  | | | Reference | | | | | | | | | | | | | | | | |  | | |  |  |
| Kiwi | (16) | 0.45 | (0.12-1.62) | 0.219 | | 1.75 | | (0.42-7.25) | | 0.443 | | | 0.80 | | | | | | (0.16-3.92) | | | | | 0.778 | | | | | |  | | |  |  |
| Any forceps | (34) | 0.51 | (0.19-1.36) | 0.177 | | 0.60 | | (0.19-1.93) | | 0.394 | | | 0.45 | | | | | | (0.12-1.68) | | | | | 0.234 | | | | | |  | | |  |  |
| **OASIS characteristics** |  |  |  |  | |  | |  | |  | | |  | | | | | |  | | | | |  | | | | | |  | | |  |  |
| OASIS classification |  |  |  |  | |  | |  | |  | | |  | | | | | |  | | | | |  | | | | | |  | | |  |  |
| 3A | (37) |  | Reference |  | |  | | Reference | |  | | | Reference | | | | | | | | | | | | | | | | |  | | |  |  |
| 3B | (43) | 0.90 | (0.28-2.90) | 0.859 | | 0.29 | | (0.07-118) | | 0.084 | | | 2.89 | | | | | | (0.61-13.63) | | | | | | 0.179 | | | | | |  | | |  |
| 3C/4 | (19) | 1.00 | (0.24-4.17) | 0.997 | | 0.17 | | (0.03-0.98) | | *0.047* | | | 2.16 | | | | | | (0.34-13.68) | | | | | | 0.413 | | | | | |  | | |  |
| Unspecified | (26) | 0.14 | (0.01-2.30) | 0.168 | | 0.40 | | (0.02-9.81) | | 0.577 | | | 18.31 | | | | | | (0.55-615.99) | | | | | | 0.105 | | | | | |  | | |  |
| OASIS repair method |  |  |  |  | |  | |  | |  | | |  | | | | | |  | | | | | |  | | | | |  | | |  |  |
| End-to-end | (55) |  | Reference |  | |  | | Reference | |  | | | Reference | | | | | | | | | | | | | | | | |  | | |  |  |
| Overlap | (41) | 0.76 | (0.27-2.12) | 0.602 | | 4.23 | | (1.14-15.77) | | *0.032* | | | 0.87 | | | | | | (0.22-3.48) | | | | | 0.846 | | | | | |  | | |  |  |
| Unspecified | (29) | 3.86 | (0.27-55.25) | 0.320 | | 0.83 | | (0.04-17.10) | | 0.904 | | | 0.08 | | | | | | (0.00-3.12) | | | | | 0.131 | | | | | |  | | |  |  |
| **Neonatal characteristics** |  |  |  |  | |  | |  | |  | | |  | | | | | |  | | | | |  | | | | | |  | | |  |  |
| Birthweight (for study birth) |  | 1.00 | (1.00-1.00) | 0.371 | | 1.00 | | (1.00-1.00) | | 0.745 | | | 1.00 | | | | | | (1.00-1.00) | | | | | 0.626 | | | | | |  | | |  |  |
|  |  |  | | | |  | | | | | | |  | | | | | | | | | | | | | | | | |  | | |  |  |
| **Characteristic (n/125)** |  | **Physical Limitations** | | | | **Social Limitations** | | | | | | | **Personal Relationships** | | | | | | | | | | | | | | | | |  | | |  |  |
|  |  | OR | 95% CI | | *p* | OR | 95% CI | | | *p* | | | OR | | | | | | | 95% CI | | | | | | *p* | | | |  | | |  |  |
| **Bowel symptoms at postnatal MHQ completion** | |  |  | |  |  | |  | | | |  |  | | | | | | |  | | | | | | | |  | | | |  | | |
| Faecal urgency |  |  |  | |  |  | |  | | | |  |  | | | | | | |  | | | | | | | |  | | | |  | | |
| Absent | (28) | ---- | ---- | | ---- | ---- | | ---- | | | | ---- | ---- | | | | | | | | ---- | | ---- | | | | | | | | |  | | |
| Present | (97) | ---- | ---- | | ---- | ---- | | ----- | | | | *----* | ---- | | | | | | | | ----- | | *----* | | | | | | | | |  | | |
| Difficulty wiping clean |  |  |  | |  |  | |  | | | |  |  | | | | |  | | | | | | | |  | | | | | |  | | |
| Absent | (73) |  | Reference | |  |  | | Reference | | | |  | Reference | | | | | | | | | | | | | | | | | | |  | | |
| Present | (52) | 4.95 | (1.22-20.11) | | *0.025* | 3.12 | | (0.52-18.79) | | | | 0.215 | 3.89 | | | | | | | (0.56-27.10) | | | | | | 0.170 | | | | | |  | | |
| Poor control of flatus |  |  |  | |  |  | |  | | | |  |  | | | | | | |  | | | | | |  | | | | | |  | | |
| Absent | (53) |  | Reference | |  |  | | Reference | | | |  | Reference | | | | | | | | | | | | | | | | | | |  | | |
| Present | (72) | 6.11 | (0.88-42.53) | | 0.068 | 3.00 | | (0.41-21.80) | | | | 0.277 | 5.98 | | | | | | | (0.63-57.03) | | | | | | 0.120 | | | | | |  | | |
| Any faecal leakage |  |  |  | |  |  | |  | | | |  |  | | | | | | |  | | | | | |  | | | | | |  | | |
| Absent | (93) |  | Reference | |  |  | | Reference | | | |  | Reference | | | | | | | | | | | | | | | | | | |  | | |
| Present | (32) | 2.50 | (0.64-9.68) | | 0.187 | 9.49 | | (1.73-52.03) | | | | *0.010* | 3.22 | | | | | | | (0.54-19.19) | | | | | | 0.199 | | | | | |  | | |
| **Maternal characteristics** |  |  |  | |  |  | |  | | | |  |  | | | | | | |  | | | | | |  | | | | | |  | | |
| Age at OASIS |  | 1.00 | (0.85-1.17) | | 0.997 | 1.00 | | (0.82-1.23) | | | | 0.951 | 1.22 | | | | | | | (0.98-1.52) | | | | | | 0.075 | | | | | |  | | |
| Vaginal interval birth(s) |  |  |  | |  |  | |  | | | |  |  | | | | | | |  | | | | | |  | | | | | |  | | |
| None | (103) |  | Reference | |  |  | | Reference | | | |  | Reference | | | | | | | | | | | | | | | | | | |  | | |
| Vaginal | (22) | 3.57 | (0.26-48.11) | | 0.338 | 7.33 | | (0.33-163.94) | | | | 0.209 | 0.72 | | | | | | | (0.04-14.21) | | | | | | 0.830 | | | | | |  | | |
| Total Parity (all birth modes) |  |  |  | |  |  | |  | | | |  |  | | | | | | |  | | | | | |  | | | | | |  | | |
| 2 | (87) |  | Reference | |  |  | | Reference | | | |  | Reference | | | | | | | | | | | | | | | | | | |  | | |
| ≥ 3 | (38) | 2.91 | (0.46-18.42) | | 0.258 | 2.76 | | (0.30-25.69) | | | | 0.373 | 11.75 | | | | | | | (1.00-138.06) | | | | | | 0.050 | | | | | |  | | |
| Mode of study birth |  |  |  | |  |  | |  | | | |  |  | | | | | | |  | | | | | |  | | | | | |  | | |
| Vaginal | (74) |  | Reference | |  |  | | Reference | | | |  | Reference | | | | | | | | | | | | | | | | | | |  | | |
| Caesarean section | (51) | 4.56 | (1.02-20.45) | | *0.048* | 7.37 | | (0.89-61.18) | | | | 0.064 | 0.90 | | | | | | | (0.14-5.61) | | | | | | 0.908 | | | | | |  | | |
| **Intrapartum characteristics** |  |  |  | |  |  | |  | | | |  |  | | | | | | |  | | | | | |  | | | | | |  | | |
| OASIS birth mode |  |  |  | |  |  | |  | | | |  |  | | | | | | |  | | | | | |  | | | | | |  | | |
| SVD | (75) |  | Reference | |  |  | | Reference | | | |  | Reference | | | | | | | | | | | | | | | | | | |  | | |
| Kiwi/ventouse | (16) | 3.30 | (0.48-22.63) | | 0.224 | 0.31 | | (0.02-5.86) | | | | 0.433 | 0.72 | | | | | | | (0.05-11.05) | | | | | | 0.814 | | | | | |  | | |
| Any forceps | (34) | 0.54 | (.012-2.45) | | 0.425 | 0.24 | | (0.03-2.09) | | | | 0.195 | 0.17 | | | | | | | (0.02-1.37) | | | | | | 0.096 | | | | | |  | | |
| **OASIS characteristics** |  |  |  | |  |  | |  | | | |  |  | | | | | | |  | | | | | |  | | | | | |  | | |
| OASIS classification |  |  |  | |  |  | |  | | | |  |  | | | | | | |  | | | | | |  | | | | | |  | | |
| 3A | (37) |  | Reference | |  |  | | Reference | | | |  | Reference | | | | | | | | | | | | | | | | | | |  | | |
| 3B | (43) | 8.27 | (0.96-71.29) | | 0.054 | 0.42 | | (0.04-4.49) | | | | 0.471 | 4.09 | | | | | | | (0.36-46.48) | | | | | | 0.256 | | | | | |  | | |
| 3C/4 | (19) | 1.00 | (0.10-9.96) | | 1.000 | 0.08 | | (0.00-1.79) | | | | 0.110 | 0.27 | | | | | | | (0.01-5.32) | | | | | | 0.393 | | | | | |  | | |
| Unspecified | (26) | 0.83 | (0.04-18.26) | | 0.905 | 807413.8 | | (0) | | | | 0.995 |  | | | | | | |  | | | | | |  | | | | | |  | | |
| OASIS repair method |  |  |  | |  |  | |  | | | |  |  | | | | | | |  | | | | | |  | | | | | |  | | |
| End-to-end | (55) |  | Reference | |  |  | | Reference | | | |  | Reference | | | | | | | | | | | | | | | | | | |  | | |
| Overlap | (41) | 0.87 | (0.17-4.49) | | 0.863 | 4.02 | | (0.44-36.99) | | | | 0.220 | 0.17 | | | | | | | (0.02-1.84) | | | | | | 0.144 | | | | | |  | | |
| Unspecified | (29) | 3.32 | (0.17-64.71) | | 0.429 | 6.42E-07 | | (0) | | | | 0.995 | 3.97 | | | | | | | (0.14-109.96) | | | | | | 0.415 | | | | | |  | | |
| **Neonatal characteristics** |  |  |  | |  |  | |  | | | |  |  | | | | | | |  | | | | | |  | | | | | |  | | |
| Birthweight (for study birth) |  | 1.00 | (1.00-1.00) | | 0.307 | 1.00 | | (1.00-1.00) | | | | 0.408 | 1.00 | | | | | | | (1.00-1.00) | | | | | | 0.310 | | | | | |  | | |
|  |  |  | | | |  | | | | | | |  | | | | | | | | | | | | | | | | |  | | |  |  |
| **Characteristic (n/125)** |  | **Emotions** | | | | **Sleep/Energy** | | | | | | | **Severity Measures** | | | | | | | | | | | | | | | | |  | | |  |  |
|  |  | OR | 95% CI | *P* | | OR | | | 95% CI | | *p* | | OR | 95% CI | | | | | | | | | | *p* | | | | | |  | | |  |  |
| **Bowel symptoms at postnatal MHQ completion** | |  |  |  | |  | | |  | |  | |  | | | |  | | | | | | |  | | | | | |  | | |  |  |
| Faecal urgency |  |  |  |  | |  | | |  | |  | |  | | | |  | | | | | | |  | | | | | |  | | |  |  |
| Absent | (28) |  | Reference |  | |  | | | Reference | |  | | Reference | | | | | | | | | | | | | | | | |  | | |  |  |
| Present | (97) | 3.56 | (0.86-14.68) | 0.079 | | 16.16 | | | (1.14-228.62) | | *0.040* | | 2.36 | | | | | (0.44-12.66) | | | | | | | 0.317 | | | | |  | | |  |  |
| Difficulty wiping clean |  |  |  |  | |  | | |  | |  | |  | | |  | | | | | | | | |  | | | | |  | | |  |  |
| Absent | (73) |  | Reference |  | |  | | | Reference | |  | | Reference | | | | | | | | | | | | | | | | |  | | |  |  |
| Present | (52) | 3.89 | (1.40-10.78) | *0.009* | | 0.98 | | | (0.19-5.02) | | 0.979 | | 5.40 | | | | | (1.71-17.02) | | | | | | | *0.004* | | | | |  | | |  |  |
| Poor control of flatus |  |  |  |  | |  | | |  | |  | |  | | | | |  | | | | | | |  | | | | |  | | |  |  |
| Absent | (53) |  | Reference |  | |  | | | Reference | |  | | Reference | | | | | | | | | | | | | | | | |  | | |  |  |
| Present | (72) | 2.09 | (0.66-6.60) | 0.210 | | 0.60 | | | (0.10-3.60) | | 0.572 | | 1.88 | | | | | (0.53-6.68) | | | | | | | 0.331 | | | | |  | | |  |  |
| Any faecal leakage |  |  |  |  | |  | | |  | |  | |  | | | | |  | | | | | | |  | | | | |  | | |  |  |
| Absent | (93) |  | Reference |  | |  | | | Reference | |  | | Reference | | | | | | | | | | | | | | | | |  | | |  |  |
| Present | (32) | 1.56 | (5.01-4.87) | 0.443 | | 5.21 | | | (1.04-26.23) | | *0.045* | | 5.54 | | | | | (1.63-18.77) | | | | | | | *0.006* | | | | |  | | |  |  |
| **Maternal characteristics** |  |  |  |  | |  | | |  | |  | |  | | | | |  | | | | | | |  | | | | |  | | |  |  |
| Age at OASIS |  | 1.01 | (0.90-1.13) | 0.851 | | 0.97 | | | (0.81-1.16) | | 0.707 | | 0.94 | | | | | (0.82-1.07) | | | | | | | 0.320 | | | | |  | | |  |  |
| Vaginal interval birth(s) |  |  |  |  | |  | | |  | |  | |  | | | | |  | | | | | | |  | | | | |  | | |  |  |
| None | (103) |  | Reference |  | |  | | | Reference | |  | | Reference | | | | | | | | | | | | | | | | | | | |  |  |
| Vaginal | (22) | 0.56 | (0.10-3.34) | 0.527 | | 1.17 | | | (0.10-13.32) | | 0.901 | | 0.42 | | | | (0.05-3.60) | | | | | | | | 0.427 | | | | |  | | |  |  |
| Parity (all birth modes) |  |  |  |  | |  | | |  | |  | |  | | | |  | | | | | | | |  | | | | |  | | |  |  |
| 2 | (87) |  | Reference |  | |  | | | Reference | |  | | Reference | | | | | | | | | | | | | | | | |  | | |  |  |
| ≥ 3 | (38) | 8.49 | (1.87-38.44) | *0.006* | | 5.72 | | | (0.91-36.16) | | 0.064 | | 3.45 | | | | | (0.71-16.85) | | | | | | | 0.126 | | | | |  | | |  |  |
| Mode of study birth |  |  |  |  | |  | | |  | |  | |  | | | | |  | | | | | | |  | | | | |  | | |  |  |
| Vaginal | (74) |  | Reference |  | |  | | | Reference | |  | | Reference | | | | | | | | | | | | | | | | |  | | |  |  |
| Caesarean section | (51) | 2.20 | (0.79-6.11) | 0.131 | | 4.77 | | | (0.90-25.17) | | 0.066 | | 2.18 | | | | (0.69-6.82) | | | | | | | | 0.182 | | | | |  | | |  |  |
| **Intrapartum characteristics** |  |  |  |  | |  | | |  | |  | |  | | | |  | | | | | | | |  | | | | |  | | |  |  |
| OASIS birth mode |  |  |  |  | |  | | |  | |  | |  | | | |  | | | | | | | |  | | | | |  | | |  |  |
| SVD | (75) |  | Reference |  | |  | | | Reference | |  | | Reference | | | | | | | | | | | | | | | | |  | | |  |  |
| Kiwi | (16) | 1.13 | (0.28-4.58) | 0.866 | | 0.29 | | | (0.02-3.87) | | 0.346 | | 1.28 | | | | (0.27-6.19) | | | | | | | | 0.755 | | | | |  | | |  |  |
| Any forceps | (34) | 0.36 | (0.11-1.19) | 0.094 | | 0.46 | | | (0.08-2.62) | | 0.383 | | 0.24 | | | | (0.06-0.96) | | | | | | | | *0.043* | | | | |  | | |  |  |
| **OASIS characteristics** |  |  |  |  | |  | | |  | |  | |  | | | |  | | | | | | |  | | | | | |  | | |  |  |
| OASIS classification |  |  |  |  | |  | | |  | |  | |  | | | |  | | | | | | |  | | | | | |  | | |  |  |
| 3A | (37) |  | Reference |  | |  | | | Reference | |  | | Reference | | | | | | | | | | | | | | | | | | | |  |  |
| 3B | (43) | 1.25 | (0.31-5.09) | 0.753 | | 1.31 | | | (0.14-12.34) | | 0.816 | | 3.63 | | | | | (0.74-17.71) | | | | | | | 0.111 | | | | |  | | |  |  |
| 3C/4 | (19) | 1.75 | (0.34-9.11) | 0.504 | | 0.83 | | | (0.04-15.52) | | 0.898 | | 3.82 | | | | | (0.57-25.52) | | | | | | | 0.166 | | | | |  | | |  |  |
| Unspecified | (26) | 2.00 | (0.11-36.11 | 0.640 | | 2009844 | | | (0) | | 0.993 | | 0.11 | | | | | (0.00-2.77) | | | | | | | 0.179 | | | | |  | | |  |  |
| OASIS repair method |  |  |  |  | |  | | |  | |  | |  | | |  | | | | | | | |  | | | | | |  | | |  |  |
| End-to-end | (55) |  | Reference |  | |  | | | Reference | |  | | Reference | | | | | | | | | | | | | | | | |  | | |  |  |
| Overlap | (41) | 0.90 | (0.26-3.13) | 0.863 | | 0.47 | | | (0.07-3.34) | | 0.451 | | 0.40 | | | | | (0.10-1.57) | | | | | | | 0.188 | | | | |  | | |  |  |
| Unspecified | (29) | 0.30 | (0.02-4.60) | 0.384 | | 1.80E-06 | | | (0) | | 0.993 | | 4.04 | | | | | (0.20-81.98) | | | | | | | 0.363 | | | | |  | | |  |  |
| **Neonatal characteristics** |  |  |  |  | |  | | |  | |  | |  | | | |  | | | | | | | |  | | | | |  | | |  |  |
| Birthweight (for study birth) |  | 1.00 | (1.00-1.00) | 0.732 | | 1.00 | | | (1.00-1.00) | | 0.875 | | 1.00 | | | | (1.00-1.00) | | | | | | | | 0.829 | | | | |  | | |  |  |
