# Supplementary material for: The impact of mode of subsequent birth after obstetric anal sphincter injury on bowel function and related quality of life: a cohort study
Source: Int Urogynecol J. 2020 Feb 24;31(11):2237–45. doi: 10.1007/s00192-020-04234-3 (PMC7561530; doi:10.1007/s00192-020-04234-3)
Supplement: Supplementary file 7 — (DOCX 16 kb) [file 192_2020_4234_MOESM7_ESM.docx]

**Supplementary Table 7**

|  |  | EAUS findings following subsequent birth, N=105 (vaginal birth = 66, caesarean section = 39) | | | | | | | |
| --- | --- | --- | --- | --- | --- | --- | --- | --- | --- |
|  |  | No anal sphincter defect, 77 (73.3) | | | | Anal sphincter defect present, 28 (26.7) | | | |
|  |  | Postnatal MHQ bowel frequency compared to antenatal MHQ bowel frequency | | | | Postnatal MHQ bowel frequency compared to antenatal MHQ bowel frequency | | | |
| Bowel function following study birth | Mode of study birth | Worsened | No change | Improved | *p* value ^♐^ | Worsened | No change | Improved | *p* value ^♐^ |
| Bowel Urgency | vaginal | 19 (31.2) | 26 (42.6) | 16 (23.2) | 0.724 | 1 (20.0) | 3 (60.0) | 1 (20.0) | 1.000 |
|  | caesarean section | 4 (25.0) | 6 (37.5) | 6 (37.5) |  | 4 (17.4) | 12 (52.2) | 7 (30.4) |  |
| Poor control of flatus | vaginal | 11 (18.0) | 34 (55.7) | 16 (23.2) | 0.238 | 2 (40.0) | 2 (40.0) | 1 (20.0) | 0.806 |
|  | caesarean section | 2 (12.5) | 6 (37.5) | 8 (50.0) |  | 5 (21.7) | 13 (56.5) | 5 (21.7) |  |
| Faecal leakage – passive only | vaginal | 1 (1.6) | 57 (93.4) | 3 (4.9) | 0.498 | 0 | 4 (80.0) | 1 (20.0) | 0.331 |
|  | caesarean section | 1 (6.3) | 14 (87.5) | 1 (6.3) |  | 1 (4.4) | 22 (95.7) | 0 |  |
| Any faecal leakage | vaginal | 8 (13.1) | 49 (80.3) | 4 (6.6) | 0.313 | 0 | 4 (80.0) | 1 (20.0) | 1.000 |
|  | caesarean section | 1 (6.3) | 12 (75.0) | 3 (18.8) |  | 1 (4.4) | 19 (82.6) | 3 (13.0) |  |
