# Supplementary material for: The impact of mode of subsequent birth after obstetric anal sphincter injury on bowel function and related quality of life: a cohort study
Source: Int Urogynecol J. 2020 Feb 24;31(11):2237–45. doi: 10.1007/s00192-020-04234-3 (PMC7561530; doi:10.1007/s00192-020-04234-3)
Supplement: Supplementary file 8 — (DOCX 18 kb) [file 192_2020_4234_MOESM8_ESM.docx]

**Supplementary Table 8**

|  |  | EAUS findings following subsequent birth, N=105 (vaginal birth = 66, caesarean section = 39) | | | | | | | |
| --- | --- | --- | --- | --- | --- | --- | --- | --- | --- |
|  |  | No anal sphincter defect, 77 (73.3) | | | | Anal sphincter defect present, 28 (26.7) | | | |
|  |  | Postnatal MHQ QoL domain score compared to antenatal MHQ QoL domain score | | | | Postnatal MHQ QoL domain score compared to antenatal MHQ QoL domain score | | | |
| MHQ QoL domain | Mode of study birth | Worsened score | No change in score | Improved score | *p* value^♐^ | Worsened score | No change in score | Improved score | *p* value^♐^ |
| General Health Perception (GHP) | vaginal | 8 (13.1) | 37 (60.7) | 16 (23.2) | 0.674 | 1 (20.0) | 3 (60.0) | 1 (20.0) | 0.280 |
|  | caesarean section | 2 (12.5) | 8 (50.0) | 6 (37.5) |  | 0 | 14 (60.9) | 9 (39.1) |  |
| Incontinence Impact (II) | vaginal | 11 (18.0) | 38 (62.3) | 12 (19.7) | 1.000 | 1 (20.0) | 4 (80.0) | 0 | 0.335 |
|  | caesarean section | 3(18.8) | 10 (62.5) | 3 (18.8) |  | 2 (8.7) | 14 (60.9) | 7 (30.4) |  |
| Role Limitations (RL) | vaginal | 6 (9.9) | 51 (83.6) | 4 (6.6) | 0.109 | 2 (40.0) | 3 (60.0) | 0 | 0.144 |
|  | caesarean section | 4 (25.0) | 10 (62.5) | 2 (12.5) |  | 1 (4.4) | 19 (82.6) | 3 (13.0) |  |
| Physical Limitations (PL) | vaginal | 1 (1.6) | 53 (86.9) | 7 (11.5) | *0.014* | 1 (20.0) | 4 (80.0) | 0 | 1.000 |
|  | caesarean section | 2 (12.5) | 9 (56.3) | 5 (31.2) |  | 3 (13.0) | 17 (73.9) | 3 (13.0) |  |
| Social Limitations (SL) | vaginal | 2 (3.3) | 55 (90.2) | 4 (6.6) | 0.400 | 1 (20.0) | 4 (80.0) | 0 | 0.658 |
|  | caesarean section | 1 (6.25) | 13 (81.3) | 2 (12.5) |  | 2 (8.7) | 19 (82.6) | 2 (8.7) |  |
| Personal Relationships (PR) | vaginal | 3 (4.9) | 54 (88.5) | 4 (6.6) | 0.590 | 1 (20.0) | 4 (80.0) | 0 | 0.459 |
|  | caesarean section | 1 (6.25) | 11 (68.8) | 4 (25.0) |  | 1 (4.4) | 21 91.3) | 1 (4.4) |  |
| Emotions (E) | vaginal | 6 (9.9) | 46 (75.4) | 9 (14.8) | 0.556 | 1 (20.0) | 3 (60.0) | 1 (20.0) | 0.367 |
|  | caesarean section | 0 | 13 (81.3) | 3 (18.8) |  | 2 (8.7) | 18 (78.3) | 3 (13.0) |  |
| Sleep/Energy (SE) | vaginal | 2 (3.3) | 54 (88.5) | 5 (8.2) | 0.522 | 0 | 5 (100.0) | 0 | 1.000 |
|  | caesarean section | 1 (6.25) | 13 (81.3) | 2 (12.5) |  | 2 (8.7) | 19 (82.6) | 2 (8.7) |  |
| Severity Measure (SM) | vaginal | 4 (6.6) | 45 (73.8) | 12 (19.7) | 0.143 | 1 (20.0) | 4 (80.0) | 0 | 1.000 |
|  | caesarean section | 2 (12.5) | 8 (50.0) | 6 (37.5) |  | 4 (17.4) | 15 (65.2) | 4 (17.4) |  |
